# High-Precision Stored-Grain Insect Pest Detection Method Based on PDA-YOLO

**DOI:** 10.3390/insects16060610

**Published:** 2025-06-10

**Authors:** Fuyan Sun, Zhizhong Guan, Zongwang Lyu, Shanshan Liu

**Affiliations:** 1 Key Laboratory of Grain Information Processing and Control, Ministry of Education, Henan University of Technology, Zhengzhou 450001, China; 2 Henan Key Laboratory of Grain Storage Information Intelligent Perception and Decision Making, Henan University of Technology, Zhengzhou 450001, China; 3 College of Information Science and Engineering, Henan University of Technology, Zhengzhou 450001, China

**Keywords:** computer vision, stored-grain insect pests, YOLO11, object detection, food security

## Abstract

Effective stored-grain insect pest detection is crucial in grain storage management to prevent economic losses and ensure food security throughout production and supply chains. Existing detection methods suffer from issues such as high labor costs, environmental interference, high equipment costs, and inconsistent performance. To address these limitations, we proposed PDA-YOLO, an improved stored-grain insect pest detection algorithm based on YOLO11n which integrates three key modules: PoolFormer_C3k2 (PF_C3k2) for efficient local feature extraction, Attention-based Intra-Scale Feature Interaction (AIFI) for enhanced global context awareness, and Dynamic Multi-scale Aware Edge (DMAE) for precise boundary detection of small targets. Trained and tested on 6200 images covering five common stored-grain insect pests (Lesser Grain Borer, Red Flour Beetle, Indian Meal Moth, Maize Weevil, and Angoumois Grain Moth), PDA-YOLO achieved an mAP@0.5 of 96.6%, mAP@0.5:0.95 of 60.4%, and *F*1 score of 93.5%, with a computational cost of only 6.9 G and mean detection time of 9.9 ms per image. These results demonstrate the advantages over mainstream detection algorithms, balancing accuracy, computational efficiency, and real-time performance. PDA-YOLO provides a reference for pest detection in intelligent grain storage management.

## Simple Summary

Pests that infest stored grain cause significant economic losses and threaten food security by consuming grain and contaminating it with waste products. Current methods for detecting these insect pests have limitations such as high labor costs, environmental interference, and expensive equipment. Our research developed a new computer vision algorithm called PDA-YOLO that can automatically detect five common types of stored-grain insect pests with high accuracy: Lesser Grain Borer, Red Flour Beetle, Indian Meal Moth, Maize Weevil, and Angoumois Grain Moth. The algorithm works by analyzing images to identify small insects against complex grain backgrounds. We designed special features that help the algorithm recognize pest shapes and distinguish them from grain debris. In our test set, our algorithm achieved detection accuracy higher than currently common algorithms and worked quickly enough for real-time monitoring. This technology provides a practical tool for grain storage managers to detect pest infestations early, thus reducing crop losses and chemical treatments. By improving pest monitoring in grain storage facilities, this algorithm helps protect our food supply and reduces the waste of vital agricultural resources.

## 1. Introduction

Food security has become a global concern with population growth expected to increase food demand by 50% by 2050 [1]. Despite continuous growth in global food production, losses during storage and transportation have become more prominent due to various factors. In Asia, approximately 6% of post-harvest losses are attributed to improper storage methods, with biological damage caused by stored-grain insect pests and fungi accounting for about 50% of total losses [2]. Stored-grain insect pests not only directly consume grain but also contaminate it through their excrement, secretions, and carcasses, producing acidic and pungent odors [3]. Additionally, pest infestations reduce the germination capacity of stored grain seeds, compromising their viability for planting purposes [4,5]. This contamination and reduced seed viability collectively diminish the nutritional value and safety of the grain while simultaneously posing health risks to consumers [6]. Beyond the direct impact on grain quality, such losses also represent a massive waste of natural resources, including water, fertilizers, energy, and land (overuse accounting for over 20% of total resource utilization) [7]. Therefore, the timely detection of pest infestations in stored-grain has become a critical measure to ensure food security and enhance the efficiency of food production.

Traditional methods for detecting stored-grain insect pests include visual inspection (bias and personal visual limitations of humans who control storage facilities), probe sampling (time consuming and labor intensive), and trapping techniques (requiring visual inspection of trapped pests) [8,9]. Modern pest detection methods offer more efficient alternatives. Acoustic detection identifies pests by capturing specific sound signals but faces background noise interference and high sensor costs [10,11,12]. Near-infrared spectroscopy (NIRS) analyzes absorption characteristics in the near-infrared spectrum, allowing for on-the-fly analysis, but it is limited by equipment costs and environmental variables [13,14]. Electronic noses detect volatile organic compounds (VOCs) produced by pests, exhibiting high sensitivity and automation potential, though performance can be influenced by environmental factors [15,16]. These limitations highlight the need for more robust detection approaches, leading to the exploration of computer vision technologies for stored-grain insect pest detection.

Computer vision technology has shown promising potential in stored-grain insect pest detection, with neural networks extracting image features and various detection architectures enabling automated pest monitoring [17,18]. Deep learning-based object detection algorithms fall into two main categories: two-stage algorithms like Faster R-CNN that offer slower processing speeds, and one-stage algorithms like Single Shot Multi-box Detector (SSD) and You Only Look Once (YOLO) that provide superior real-time performance by integrating region proposal and classification [19,20,21,22]. Recent studies have further optimized one-stage algorithms for stored-grain insect pest detection. For instance, Lyu et al. [23] enhanced SSD with Top-Down feature fusion and K-means clustering to improve small-sized pest detection, addressing challenges through specialized prior bounding boxes and multi-level feature integration. Zhao et al. [24] proposed AC-YOLO, an enhanced YOLOv7 algorithm that integrates multiple attention mechanisms and Efficient Complete Intersection over Union (ECIoU) to achieve high-precision detection of 12 stored-grain insect pest species in complex backgrounds. Similarly, Li et al. [25] developed YOLO-TP, a lightweight model based on YOLOv8n that incorporates improved modules and an optimized loss function to achieve high-precision detection and counting of *Lasioderma serricorne* while reducing model parameters and computational requirements. Chen et al. [26] proposed an automatic inspection system for stored-grain insect pest detection using YOLOv4, which consists of a deep learning model embedded in a mobile car with a camera and supplementary light that navigates on granary surfaces to detect and count pests in real time. Badgujar et al. [27] developed an integrated system for real-time stored-grain insect pest detection using deep learning, which combines an RGB camera with YOLOv5 models to identify six common pest species across various platforms, including mobile devices, for use in warehouses and food facilities. These advancements underscore the growing effectiveness of one-stage object detection algorithms in addressing the unique challenges of stored-grain insect pest detection.

Despite the progress made in previous studies, existing object detection algorithms for stored-grain insect pest detection still face three key challenges: (1) Adult stored-grain insect pests are typically only a few millimeters to centimeters in length, occupying a very small number of pixels in high-resolution images. This limited visual information noticeably reduces detection accuracy and robustness. (2) The surface texture and color of grain are often similar to the color and morphology of stored-grain insect pests, leading to false positives and missed detections. (3) Existing object detection algorithms often struggle to achieve an optimal balance between detection accuracy, computational efficiency, and real-time performance. This imbalance presents challenges for practical applications requiring high precision while maintaining operational efficiency. (4) Stored-grain insect pests often conceal themselves within grain masses or among kernels, requiring sampling or trapping methods to extract them for direct observation and subsequent detection. To address these challenges, this study aimed to develop a high-precision, computationally efficient algorithm specifically designed for the real-time detection of stored-grain insect pests captured in collection bottles of monitoring traps within wheat granaries. We propose a novel approach based on the YOLO11n framework with strategic architectural modifications to enhance feature representation and object boundary delineation capabilities.

The main contributions of this study are as follows:

1. We propose PDA-YOLO, an improved algorithm based on YOLO11n specifically optimized for small target size, complex backgrounds, and real-time detection requirements in stored-grain insect pest presence detection and classification, achieving an optimal balance between detection accuracy, computational efficiency, and real-time performance.
2. We introduce the Dynamic Multi-scale Aware Edge (DMAE) module, which adaptively enhances edge features across multiple scales while dynamically adjusting branch weights based on input complexity, enabling the precise delineation of small insect pest boundaries and further improving detection accuracy.

## 2. Materials and Methods

### 2.1. YOLO11

YOLO11, developed by Ultralytics [28], is a state-of-the-art object detection algorithm that optimally balances speed and accuracy. Its architecture consists of three primary components: (1) Backbone network: Extracts fundamental features using a cross-stage partial separation strategy that mitigates gradient vanishing and accelerates convergence by passing some features directly to the neck while continuing to process the remainder. (2) Neck network: Performs feature fusion through multi-path feature aggregation to effectively integrate features from different layers, enhancing multi-scale object detection capabilities. (3) Head network: Focuses on localization and classification by decoding the multi-scale feature maps to generate detection boxes and class predictions.

**Figure 1:**
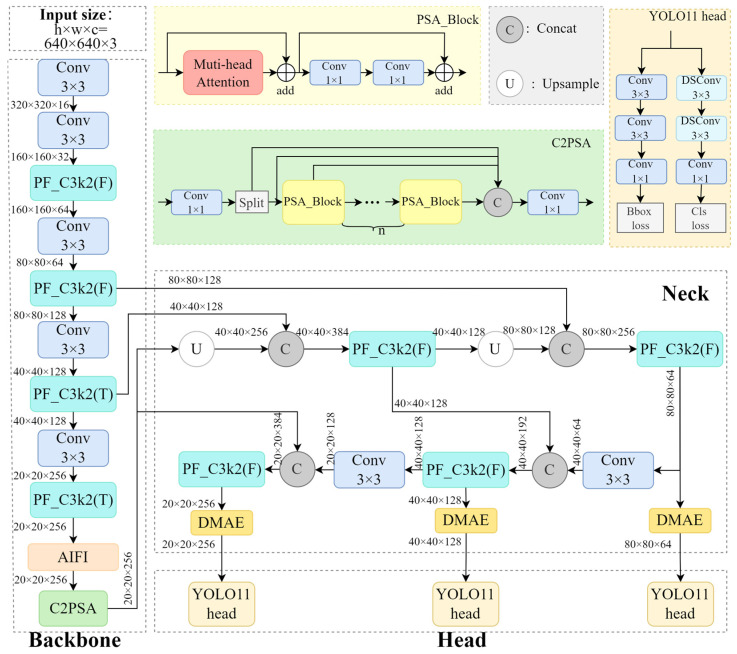
Diagram of the PDA-YOLO network architecture. In the PF_C3k2 module, T indicates that the built-in parameter is set to “true”, while F indicates that the built-in parameter is set to “false”.

Based on network width, depth, and maximum channel count, YOLO11 can be categorized into five versions from smallest to largest: n, s, m, l, and x. To achieve an optimal balance between accuracy, computational cost, and detection speed, this study selected the smallest version, YOLO11n, as the baseline model. As illustrated in Figure 1, we proposed PDA-YOLO by incorporating strategic modifications to YOLO11n. Specifically, we optimized all C3k2 modules in the network to PF_C3k2, replaced the Spatial Pyramid Pooling-Fast (SPPF) module with an AIFI module, and additionally introduced our proposed DMAE module into the neck network. These architectural enhancements purposely address the challenges of detecting tiny stored-grain insect pests against complex backgrounds, as will be detailed in subsequent sections.

### 2.2. Improved C3k2 Module

PoolFormer, as a variant of the transformer, achieves efficient interaction between tokens through simple pooling operations [29,30]. This design reduces computational complexity while retaining strong feature extraction capabilities. Meanwhile, the C3k2 module in YOLO11 offers flexible feature processing through its dynamic path-switching mechanism, where switching its built-in parameters between true and false enables adaptive feature handling for different detection scenarios. Building on the advantages of PoolFormer and the characteristics of the C3k2 module, we designed the PoolFormer_C3k2 (PF_C3k2) module to optimize the C3k2 module.

**Figure 2:**
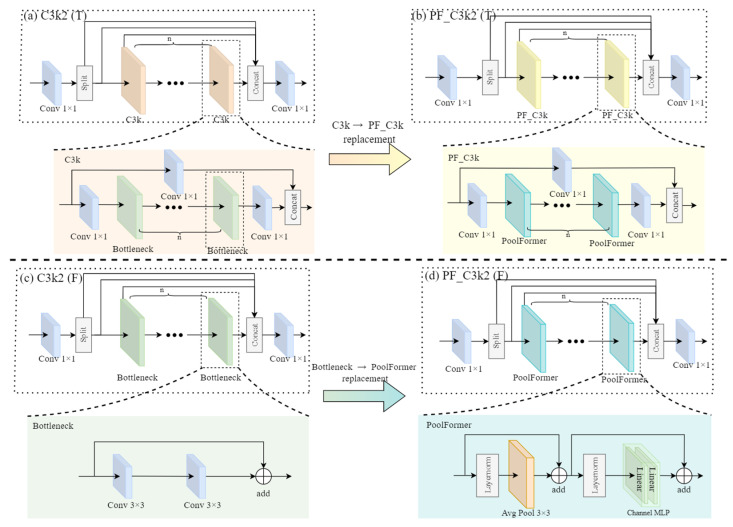
Diagram of the C3k2 and PF_C3k2 module structure. In the network architecture employed in this study, n was set to 1. T indicates that the built-in parameter is set to “true”, while F indicates that the built-in parameter is set to “false”.

As shown in Figure 2, in the PoolFormer architecture, LayerNorm is first applied to normalize the input features along the channel dimension for each sample, ensuring the stability of feature distributions across different dimensions. Then, average pooling is employed to calculate the mean of feature values within local neighborhoods, aggregating fine-grained pixel or feature information. This aggregation method effectively captures local contextual information and integrates features from adjacent regions into more representative expressions. Moreover, since average pooling does not require parameter learning, this design reduces computational resource consumption. To address the gradient vanishing problem, residual connections are introduced into the PoolFormer module. These connections improve the training stability of deep networks while preserving fine-grained information from the input features. After the pooling operation, the output features are passed through a multilayer perceptron (MLP) for nonlinear transformation, further enhancing feature representation capabilities.

In the PF_C3k2 module, when the built-in parameter is set to “true”, features are processed through the PoolFormer_C3k(PF_C3k) pathway, which replaces the conventional C3k in the original module (Figure 2a,b). This substitution enhances local feature representation through PoolFormer’s efficient token interaction, enabling a more effective detection of subtle pest features against complex grain backgrounds. This pathway is particularly suitable for scenarios requiring fine-grained discrimination of visually similar objects. Conversely, when the parameter is set to “false”, features flow through the PoolFormer pathway, which replaces the original Bottleneck component (Figure 2c,d). Compared to the conventional Bottleneck, PoolFormer introduces superior global context modeling capabilities with fewer parameters, capturing long-range dependencies that are crucial for understanding spatial relationships in pest detection images. This lightweight pathway is especially effective for enhancing global feature awareness while maintaining computational efficiency, making it ideal for resource-constrained real-time detection applications.

### 2.3. The Proposed DMAE Module

Edge information constitutes a fundamental visual cue that delineates object boundaries and shapes, providing essential spatial context for precise localization in object detection frameworks. This boundary information is particularly crucial for differentiating small objects, such as stored-grain insect pests, from complex backgrounds, where subtle edge details frequently determine detection efficacy. Despite this significance, the baseline YOLO11 architecture lacks dedicated modules for enhancing object edge features, thus potentially constraining detection accuracy. To address this limitation, we proposed the Dynamic Multi-scale Aware Edge (DMAE) module, which adaptively enhances edge features across multiple scales while dynamically adjusting branch weights based on input complexity. This approach improves bounding box precision and small-sized pest detection performance.

**Figure 3:**
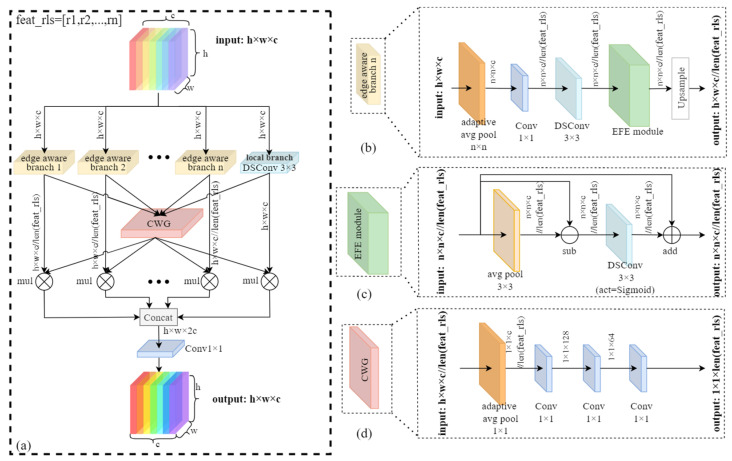
Diagram of the DMAE module we proposed. (**a**) Overall DMAE module architecture; (**b**) Edge-aware branch structure; (**c**) EFE module structure; (**d**) CWG module structure. In the figure, h represents height, w represents width, and c represents the number of channels of the feature maps. The values in feat_rls = [r1, r2, …, rn] represent the feature map resolutions for each edge-aware branch, from the first to the nth.

As illustrated in Figure 3a, the DMAE module comprises several key components organized in a parallel multi-branch architecture. The input feature map is processed through multiple parallel branches, including a local branch and several edge-aware branches operating at different scales. In each edge-aware branch, as depicted in Figure 3b, features undergo downsampling to various spatial resolutions through adaptive average pooling, where the resolutions for each branch are defined by feat_rls = [r_1_, r_2_, …, r_n_], representing the feature map resolutions from the first to the nth edge-aware branch. Specifically, in this study, feat_rls was set to [3, 6, 9, 12], corresponding to the target dimensions for adaptive pooling. This is followed by dimensionality reduction via a 1 × 1 convolution and feature extraction using a 3 × 3 DSConv. These multi-scale features then pass through the Edge Feature Enhancement (EFE) module, illustrated in Figure 3c, which is designed to accentuate boundary information in feature maps. The EFE module operates by computing the differential between the original feature map and its average-pooled version, thereby highlighting high-frequency components that predominantly correspond to edges, as formulated in Equation (1):

(1) $$F_{edge}=F_{in}-avgpool_{3\times 3}(F_{in})$$

where $F_{in}$ represents the input features of the module; $F_{edge}$ represents the edge features.

This edge representation undergoes refinement through a DSConv with a sigmoid activation function to normalize the edge response, as formulated in Equation (2):

(2) $$F_{out}=F_{in}+\sigma (DSConv_{3\times 3}(F_{edge}))$$

where σ represents the Sigmoid activation function. Subsequently, the output of the activation function is added to the input features via a residual connection, thereby preserving the original feature information.

The local branch processes the input feature map directly through a 3 × 3 DSConv, providing fine-grained local details that complement the multi-scale edge information. DSConv is employed throughout the module in place of standard convolution to minimize computational complexity while preserving representational capacity, rendering the module suitable for real-time detection applications. The outputs from both edge-aware branches and the local branch are processed by the Complexity-based Weight Generator (CWG), as shown in Figure 3d, which dynamically assesses input feature complexity to allocate appropriate weights to different branches. The CWG utilizes a lightweight network architecture comprising adaptive average pooling followed by a sequence of convolution layers that generate branch-specific weights. These weights are applied to each branch’s output, enabling the module to adaptively emphasize the most informative scales according to specific input characteristics, as illustrated in Equations (3) and (4):

(3) $$F_{local}=W_{0}\times Local\_branch(x)$$

(4) $$F_{i}=W_{i}\times Edge\_branch_{i}(x),i\in [1,n]$$

where x represents the input feature; $W_{0}$ represents the weight for the local branch; $F_{local}$ denotes the output of the local branch; $W_{i}$ represents the weight for the *i*th edge-aware branch, and $F_{i}$ denotes the output of the *i*th edge-aware branch.

The outputs from all branches undergo concatenation and processing through a final 1 × 1 convolution to integrate multi-scale edge-enhanced features with local information, as formulated in Equation (5):

(5) $$F_{c}=Conv_{1\times 1}(Concat[F_{local},F_{1},F_{2},\ldots ,F_{n}])$$

where $F_{c}$ represents the final output of the DMAE module. This comprehensive feature representation enhances the model’s capacity to precisely delineate pest boundaries against complex grain backgrounds, thereby improving detection accuracy for small, visually similar targets.

### 2.4. AIFI Module

**Figure 4:**
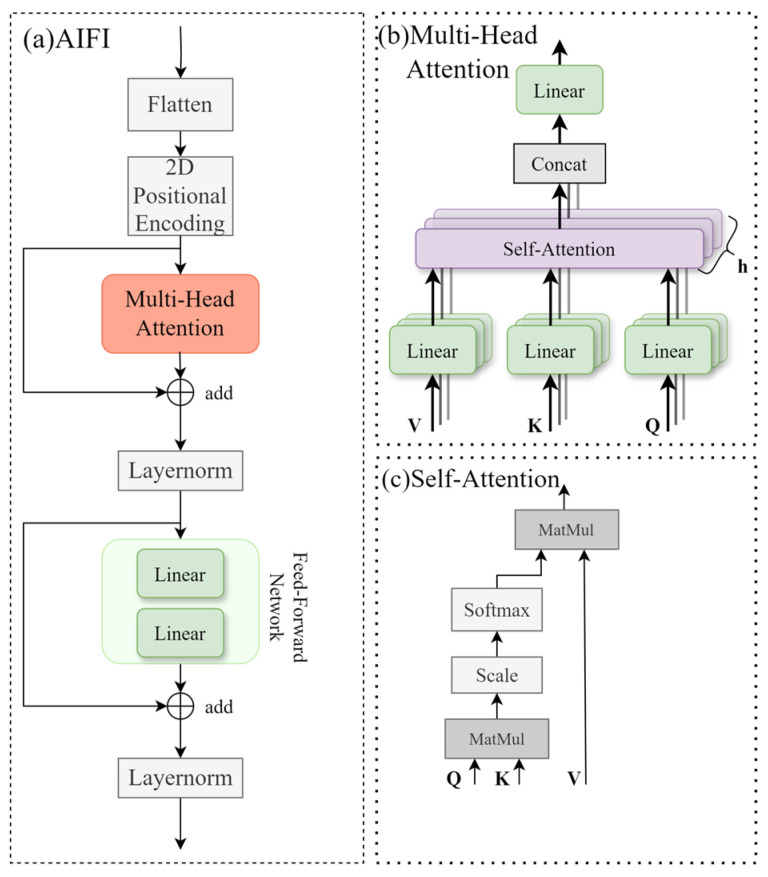
Illustration of the AIFI module. (**a**) Overall AIFI module structure. (**b**) Multi-head attention mechanism. (**c**) Self-Attention computation process.

In the context of stored-grain insect pest identification within grain storage management scenarios, pest occlusion and complex backgrounds often make it challenging for detection models to accurately recognize targets. These factors increase the demand for enhanced feature representation capabilities in the detection model. The SPPF module in the YOLO11 backbone network has insufficient ability to utilize global contextual information when processing fine-grained features and complex backgrounds. To address these issues, this study replaces the SPPF module in the YOLO11n model with the Attention-based Intra-Scale Feature Interaction(AIFI) module to enhance the network’s ability to perceive features in complex backgrounds [31]. As shown in Figure 4a, the AIFI module first flattens the input features into a one-dimensional sequence to adapt them to the input format required by transformers. Next, 2D sinusoidal position encoding is added to the features to explicitly represent spatial relationships, helping the model better capture global contextual information within target regions. As illustrated in Figure 4b,c, the module then employs a multi-head attention mechanism to calculate the correlations between features, dynamically adjusting the weights of different regions. This enhances the model’s focus on critical regional features. The attention weight calculation formula is as follows:

(6) $$Attention(Q,K,V)=\mathrm{softmax}(\frac{QK^{T}}{\sqrt{d_{k}}})V$$

where Q, K, and V represent the query, key, and value matrices, respectively. $K^{T}$ denotes the transpose of matrix K. The term $\sqrt{d_{k}}$ is a scaling factor used to prevent the similarity values from becoming excessively large, ensuring numerical stability during the computation of the attention weights.

The output features generated by the multi-head attention mechanism are added to the original input features through residual connections, followed by normalization to achieve feature fusion. This operation not only effectively mitigates the gradient vanishing problem in deep networks but also preserves the original feature information. Subsequently, the features processed through multi-head attention and normalization are passed to a MLP for further nonlinear feature extraction. The MLP consists of two fully connected layers: the first layer expands the feature dimension to a higher-dimensional latent space, and the second layer reduces it back to the original feature dimension. Finally, the output features of the MLP are added to the original input features again via residual connections and normalized, resulting in the final output features.

## 3. Materials and Implementation Details

### 3.1. Stored-Grain Insect Pest Dataset

**Figure 5:**
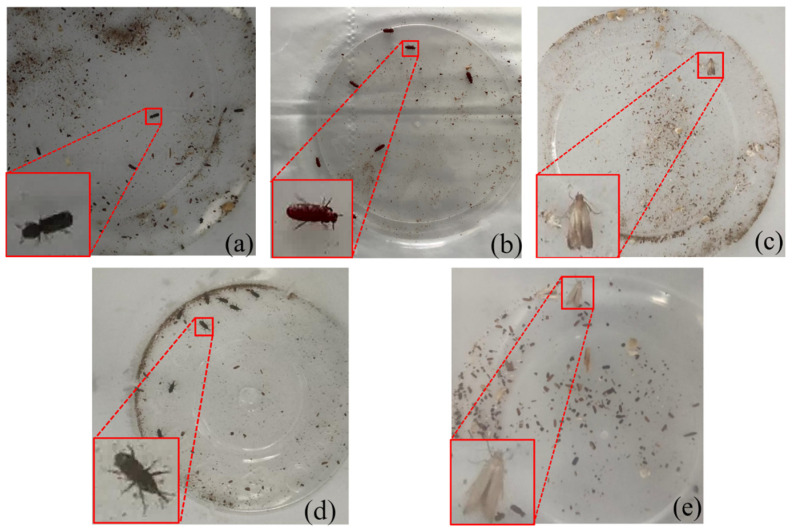
Pest samples from stored-grain insect pest dataset. (**a**) *Rhyzopertha dominica* (Lesser Grain Borer, LGB); (**b**) *Tribolium castaneum* (Red Flour Beetle, RFB); (**c**) *Plodia interpunctella* (Indian Meal Moth, IMM); (**d**) *Sitophilus zeamais* (Maize Weevil, MW); (**e**) *Sitotroga cerealella* (Angoumois Grain Moth, AGM).

This study utilized a custom dataset. Five species of common adult stored-grain insect pests were obtained from the College of Food Science and Engineering in Henan University of Technology, including *Rhyzopertha dominica* (Lesser Grain Borer, LGB), *Tribolium castaneum* (Red Flour Beetle, RFB), *Plodia interpunctella* (Indian Meal Moth, IMM), *Sitophilus zeamais* (Maize Weevil, MW), and *Sitotroga cerealella* (Angoumois Grain Moth, AGM) (Figure 5). To simulate the environment within collection bottles of monitoring traps, each species was placed in plastic collection bottles. The collection bottles were made of transparent plastic material with a cylindrical shape featuring an open top (no cap) and a circular bottom with a radius of 4 cm. Each bottle contained a small amount of wheat kernels and wheat debris to provide a realistic grain storage environment. The room temperature was maintained at 25–30 °C during image acquisition, ensuring the stored-grain insect pests remained active under these conditions. The image acquisition was conducted over two consecutive days on 19 and 20 June 2024, between 3:00 PM and 5:00 PM each day. Images were captured every 3 s, and after cropping irrelevant areas, each image had a resolution of 1100 × 1080 pixels. For each pest species, 400 images were collected, totaling 2000 images, with each image containing 1–8 pest specimens.

**Figure 6:**
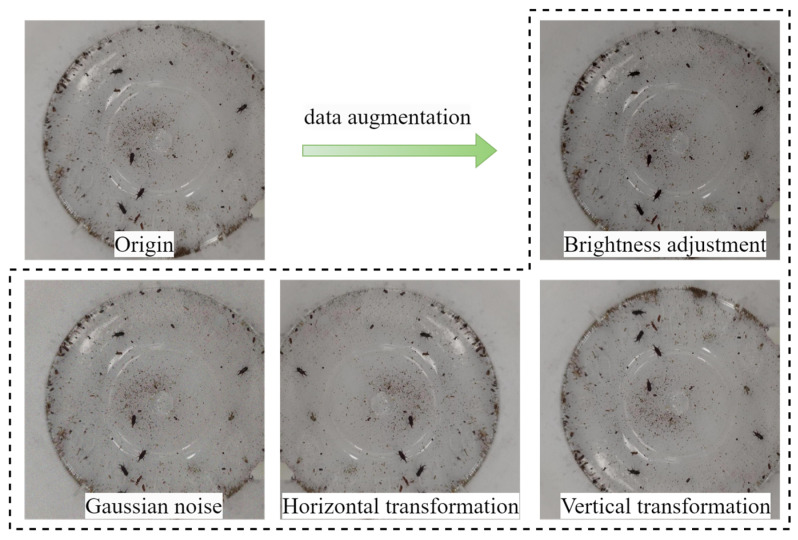
Examples of data augmentation.

To enhance model robustness, we first partitioned the 2000 original images into training, validation, and testing sets using a 7:1:2 ratio. Then, we applied data augmentation techniques to the training set, including horizontal flipping, vertical flipping, brightness reduction, and Gaussian noise addition, using the augmented images as the new training set (Figure 6). This augmentation process expanded the dataset to 6200 images, with 5600, 200, and 400 images in the training, validation, and testing sets respectively, containing a total of 31,352 pest instances, which were then manually annotated using the LabelImg software (version 1.8.6) (Table 1). The minimum enclosing rectangle method was employed for annotation, and the process was supervised by grain industry experts to ensure annotation accuracy.

**Table 1:** Statistical information of the stored-grain insect pest dataset.

Index	Species (Body Length)	Images	Training Instances	Validation Instances	Test Instances	Total Instances
1	LGB (2.3–3.0 mm)	1240	4920	161	314	5395
2	RFB (2.3–4.4 mm)	1240	6944	234	470	7648
3	IMM (8.0–10.0 mm)	1240	3832	160	367	4359
4	MW (2.5–4.5 mm)	1240	7144	136	442	7722
5	AGM (4.0–6.0 mm)	1240	5580	201	447	6228

### 3.2. Experimental Setup

All experiments in this study were conducted on a workstation equipped with an Intel(R) Xeon(R) Gold 6342 CPU operating at 2.80GHz with 32GB RAM and an NVIDIA GeForce RTX 3090 GPU with 24GB VRAM. The software environment consisted of Ubuntu 22.04 as the operating system, with CUDA 12.1 and CuDNN 8.9.0 for GPU acceleration. The programming environment utilized Python 3.11.8 and PyTorch 2.2.2 for model implementation. The hyperparameter settings used in this study are shown in Table 2, while other hyperparameters were kept at their default values.

**Table 2:** Experimental hyperparameter settings.

Parameters	Values
Epochs	150
Batch size	16
Learning rate	0.01
Optimizer	SGD
Image size	640 × 640
Momentum	0.937
Weight decay	0.0005

### 3.3. Evaluation Metrics

In this study, mean Average Precision@0.5 (mAP@0.5), mean Average Precision@0.5:0.95 (mAP@0.5:0.95), *F*1 score, Floating Point Operations (FLOPs), and mean Detection time (*mD_t_*) were selected as performance evaluation metrics. mAP@0.5 represents the Average Precision (AP) across all classes at an Intersection over Union (IoU) threshold of 0.5. mAP@0.5:0.95 calculates the AP across all classes over IoU thresholds ranging from 0.5 to 0.95 with a step size of 0.05. This metric comprehensively evaluates the detection accuracy of the model under various IoU thresholds. *F*1 score represents the harmonic mean of precision and recall, providing a balanced measure of the model’s accuracy that accounts for both false positives and false negatives. FLOPs quantify the computational complexity of the model by measuring the number of floating-point operations required to process a single image, expressed in billions. The *mD_t_* measures the average time interval, in milliseconds (ms), between image input and detection output.

The mathematical formulations of the metrics used in this study are

(7) $$mAP=\frac{1}{C}\sum_{C=1}^{C}AP_{C}$$

(8) $$AP=\int_{0}^{1}P(R)dR$$

(9) $$F1\;score=\frac{2\times P\times R}{P+R}$$

where precision P and recall R are defined as

(10) $$P=\frac{TP}{TP+FP}$$

(11) $$R=\frac{TP}{TP+FN}$$

and the *mD_t_* is

(12) $$mD_{t}=\frac{1}{n}\sum_{i=1}^{n}t_{i}$$

In these equations TP denotes the number of true-positive predictions, FP the false positives, FN the false negatives, C the total number of classes, $t_{i}$ the inference time for the *i*th image, and n the total number of test images.

## 4. Results

### 4.1. Ablation Study

To evaluate the specific impact of different modules on model performance, we conducted a series of ablation studies. These experiments systematically introduced the PF_C3k2 module, AIFI module, and DMAE module, comparing model performance across various configurations. Throughout the ablation experiments, the experimental platform configuration and hyperparameter settings remained consistent.

**Figure A1:**
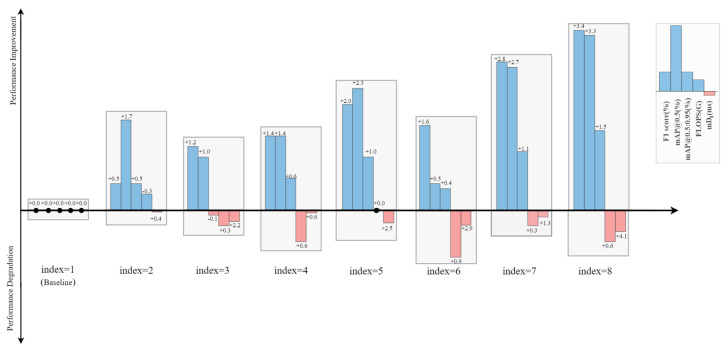
Performance gain visualization of ablation experiments. In the figure, blue bars indicate metrics that have improved compared to the baseline model, while red bars represent metrics that have declined relative to the baseline. The greater the difference from the baseline model, the taller the bar height.

As shown in Table 3 and Figure A1, where ‘√’ indicates the adoption of the corresponding method, the introduction of the PF_C3k2 module increased the mAP@0.5 from 93.3% to 95.0%, mAP@0.5:0.95 from 58.9% to 59.4%, and *F*1 score from 90.1% to 90.6%, while reducing FLOPs from 6.3 G to 6.0 G. However, the *mD_t_* increased from 5.8 ms to 6.2 ms. These improvements can be attributed to PoolFormer’s effective fusion of local information, which strengthened feature representation capabilities, while its lightweight design reduced computational resource consumption. When replacing the SPPF module in YOLO11n with the AIFI module, mAP@0.5 increased from 93.3% to 94.3%, *F*1 score increased from 90.1% to 91.3%, and mAP@0.5:0.95 remained relatively stable at 58.8% compared to the baseline. However, both computational complexity and inference time increased, with FLOPs rising to 6.6 G and *mD_t_* to 8.0 ms. This trade-off occurred because the introduction of the multi-head attention mechanism, while improving detection performance, resulted in an additional computational overhead. The integration of the DMAE module resulted in an mAP@0.5 of 94.7%, an *F*1 score of 91.5%, and an mAP@0.5:0.95 of 59.5%, with computational complexity increasing to 6.9 G and *mD_t_* to 6.4 ms. This improvement can be attributed to the module’s ability to enhance edge features through multi-scale aware processing, which is particularly effective for delineating the boundaries of small-sized pest targets against complex grain backgrounds. The dynamic branch weighting mechanism adaptively focuses on the most informative scales based on input complexity, further improving detection accuracy. Finally, the integrated PDA-YOLO, incorporating all three improvements, achieved the best performance among all configurations, with mAP@0.5 reaching 96.6%, mAP@0.5:0.95 increasing to 60.4%, and the highest *F*1 score of 93.5%. The computational requirements remained relatively modest at 6.9 G, although *mD_t_* increased to 9.9 ms. Comprehensive performance analysis indicates that PDA-YOLO, combining multiple improvement modules, represents an optimal solution for high-precision real-time stored-grain insect pest detection.

**Table 3:** Ablation study experimental data.

Index	PF_C3k2	DMAE	AIFI	*F*1 Score (%)	mAP@0.5 (%)	mAP@0.5:0.95 (%)	FLOPs (G)	*mD_t_* (ms)
1				90.1	93.3	58.9	6.3	**5.8**
2	√			90.6	95.0	59.4	**6.0**	6.2
3			√	91.3	94.3	58.8	6.6	8.0
4		√		91.5	94.7	59.5	6.9	6.4
5	√		√	92.1	95.6	59.9	6.3	8.3
6		√	√	91.7	93.8	59.3	7.2	8.7
7	√	√		92.9	96.0	60.0	6.6	7.1
8	√	√	√	**93.5**	**96.6**	**60.4**	6.9	9.9

The bold values indicate the best performance for each evaluation metric across different module combinations.

### 4.2. Comparison with Mainstream Algorithms

To comprehensively evaluate PDA-YOLO’s performance in stored-grain insect pest detection, we conducted comparative experiments against mainstream object detection algorithms, including Faster R-CNN [20], SSD [21], CenterNet [32], RT-DETR [31], YOLOv8n [28], YOLOv9t [33], YOLOv10n [34], YOLO11n [28], and Mamba-YOLO [35]. All algorithms underwent testing under identical experimental conditions.

#### 4.2.1. Comparative Analysis of Overall Performance Metrics

**Figure A2:**
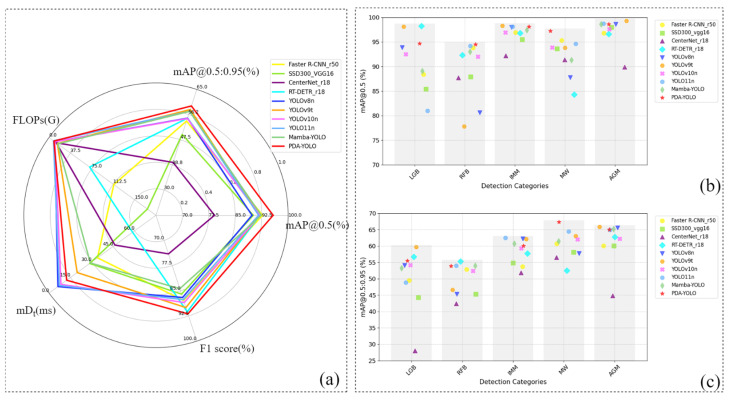
Performance comparison chart with mainstream algorithms. (**a**) Radar chart comparing performance metrics of mainstream algorithms. (**b**) Comparative analysis of category-wise mAP@0.5 across mainstream algorithms. (**c**) Comparative analysis of category-wise mAP@0.5:0.95 across mainstream algorithms.

As illustrated in Table 4 and Figure A2a, PDA-YOLO demonstrates superior performance in both detection accuracy and computational efficiency. Compared to YOLO11n, PDA-YOLO achieves improvements in mAP@0.5 from 93.3% to 96.6%, mAP@0.5:0.95 from 58.9% to 60.4%, and *F*1 score from 90.1% to 93.5% with only a minor increase in computational cost to 6.9 G. While PDA-YOLO’s *mD_t_* of 9.9 ms exceeds YOLO11n’s 5.8 ms, this trade-off is justified by the significant accuracy gains. This balance makes it ideal for stored-grain insect pest detection needs.

**Table 4:** Comparative experimental data with mainstream algorithms.

Algorithms	*F*1 Score (%)	mAP@0.5 (%)	mAP@0.5:0.95 (%)	FLOPs (G)	*mD_t_* (ms)
Faster R-CNN_r50	90.3	94.2	56.2	90.9	27.2
SSD300_vgg16	88.8	92.1	52.5	138.0	22.8
CenterNet_r18	79.2	83.3	44.7	10.2	37.0
RT-DETR_r18	93.0	93.6	57.0	57.0	46.9
YOLOv8n	89.6	91.8	57.0	8.1	**5.0**
YOLOv9t	92.0	93.5	59.4	11.7	15.8
YOLOv10n	90.8	93.6	56.9	8.3	6.7
YOLO11n	90.1	93.3	58.9	**6.3**	5.8
Mamba-YOLO	87.5	93.9	58.9	12.3	23.2
PDA-YOLO(Ours)	**93.5**	**96.6**	**60.4**	6.9	9.9

The bold values highlight the best performance for each evaluation metric across all tested algorithms.

Compared to other YOLO variants, PDA-YOLO achieves better accuracy metrics while maintaining minimal computational demands. Specifically, PDA-YOLO outperforms YOLOv8n, YOLOv9t, and YOLOv10n in mAP@0.5 by margins of 4.8%, 3.1%, and 3.0%, respectively. For mAP@0.5:0.95, our algorithm similarly surpasses these competitors by 3.4%, 1.0%, and 3.5%, respectively. Moreover, PDA-YOLO’s computational complexity of 6.9 G is considerably lower than YOLOv9t at 11.7 G, confirming its lightweight architecture efficiency. Although PDA-YOLO operates at a higher *mD_t_* compared to YOLOv8n at 5.0 ms and YOLOv10n at 6.7 ms, it still meets the real-time requirements for practical applications. Notably, PDA-YOLO shows better performance than the State Space Model-based Mamba-YOLO across all evaluation metrics. In stored-grain insect pest detection tasks, Mamba-YOLO yields inferior results with only 93.9% mAP@0.5 and 58.9% mAP@0.5:0.95, compared to PDA-YOLO’s 96.6% and 60.4%, respectively. Additionally, Mamba-YOLO’s computational complexity of 12.3 G exceeds PDA-YOLO’s efficient 6.9 G, while its *mD_t_* reaches 23.2 ms, more than twice that of PDA-YOLO at 9.9 ms. These metrics clearly show that despite theoretical advantages of SSM-based architectures, PDA-YOLO offers a more practical solution for stored-grain insect pest detection.

Regarding traditional detection frameworks, PDA-YOLO reveals clear advantages. The evaluation metrics indicate that PDA-YOLO fully outperforms Faster R-CNN, SSD300_vgg16, and CenterNet_r18 across all performance indicators. Specifically, PDA-YOLO’s mAP@0.5 is 2.4% to 13.3% higher, mAP@0.5:0.95 is 4.2% to 15.7% higher, and *F*1 score is 3.2% to 14.3% higher than these traditional algorithms. These improvements are especially noteworthy given PDA-YOLO’s computational efficiency of 6.9 G, which is markedly lower than other algorithms, merely 5.00% to 67.6% of traditional algorithms’ computational requirements, while offering significantly faster inference speeds, 12.9 ms to 27.1 ms faster.

Compared to the transformer-based detection method RT-DETR_r18, PDA-YOLO achieves a 3.0% higher mAP@0.5, 3.4% higher mAP@0.5:0.95, and 0.5% higher *F*1 score, while requiring only 6.9 G computational resources compared to RT-DETR_r18′s 57.0 G. It also offers an *mD_t_* of 9.9 ms compared to RT-DETR_r18′s 46.9 ms, providing a significantly more efficient solution for practical applications.

#### 4.2.2. Category-Wise Detection Performance Analysis

Further analysis of detection performance across different pest categories (Table 5, Figure A2b,c), reveals that PDA-YOLO achieves high accuracy across all stored-grain insect pest categories, with mAP@0.5 exceeding 94% and a minimum mAP@0.5:0.95 of 54.0% for all classes. These results demonstrate that our improvements significantly enhance feature extraction and complex background processing capabilities. Compared to YOLO11n, PDA-YOLO shows remarkable improvements in LGB and MW category detection, with mAP@0.5 increases of 13.7% and 2.6% respectively, while maintaining high accuracy for RFB, IMM, and AGM categories. Notably, while YOLOv9t achieves high detection accuracy for most categories, it exhibits performance limitations in RFB detection with mAP@0.5 and mAP@0.5:0.95 of only 77.8% and 46.6%, respectively. When examining RT-DETR_r18′s performance across different pest categories, it demonstrates excellent detection capability for LGB with mAP@0.5 reaching 98.2% and mAP@0.5:0.95 achieving 56.7%, outperforming even YOLO11n in LGB detection. However, RT-DETR_r18 exhibits significantly weaker performance in detecting MW specimens, with mAP@0.5 of only 84.3% and mAP@0.5:0.95 of merely 52.5%, which is notably lower than PDA-YOLO’s 97.2% and 67.3%, respectively. In contrast, PDA-YOLO displays more balanced and consistent performance across different pest species.

**Table 5:** mAP@0.5 (I) and mAP@0.5:0.95 (II) performance data of mainstream algorithms in detecting various pest insect species: Lesser Grain Borer (LGB), Red Flour Beetle (RFB), Indian Meal Moth (IMM), Maize Weevil (MW), and Angoumois Grain Moth (AGM).

Algorithms	Pest Insect Species									
	LGB		RFB		IMM		MW		AGM	
	I	II	I	II	I	II	I	II	I	II
Faster R-CNN_r50	88.4	49.5	93.8	52.9	96.9	57.6	95.3	60.9	96.8	60.1
SSD300_vgg16	85.4	44.3	87.9	45.3	95.5	54.8	93.6	58.1	98.0	60.1
CenterNet_r18	55.2	28.0	87.7	42.4	92.2	51.8	91.4	56.5	89.9	44.8
RT-DETR_r18	**98.2**	56.7	92.3	55.3	96.8	57.7	84.3	52.5	96.6	62.8
YOLOv8n	93.9	54.1	80.6	45.3	98.0	62.2	87.8	57.7	98.6	65.6
YOLOv9t	98.1	**59.7**	77.8	46.6	**98.3**	62.1	93.8	63.0	**99.3**	**65.8**
YOLOv10n	92.5	54.2	92.0	52.4	96.9	59.3	93.9	62.0	97.6	62.2
YOLO11n	81.0	48.9	94.2	**54.0**	98.1	**62.5**	94.6	64.4	98.7	64.9
Mamba-YOLO	89.1	53.2	93.0	**54.0**	97.4	60.7	91.3	61.5	98.6	65.2
PDA-YOLO(Ours)	94.7	55.5	**94.5**	**54.0**	98.1	60.1	**97.2**	**67.3**	98.6	65.0

The bold values represent the highest mAP for each pest category.

### 4.3. Analysis of Stored-Grain Insect Pest Detection Results

**Figure 7:**
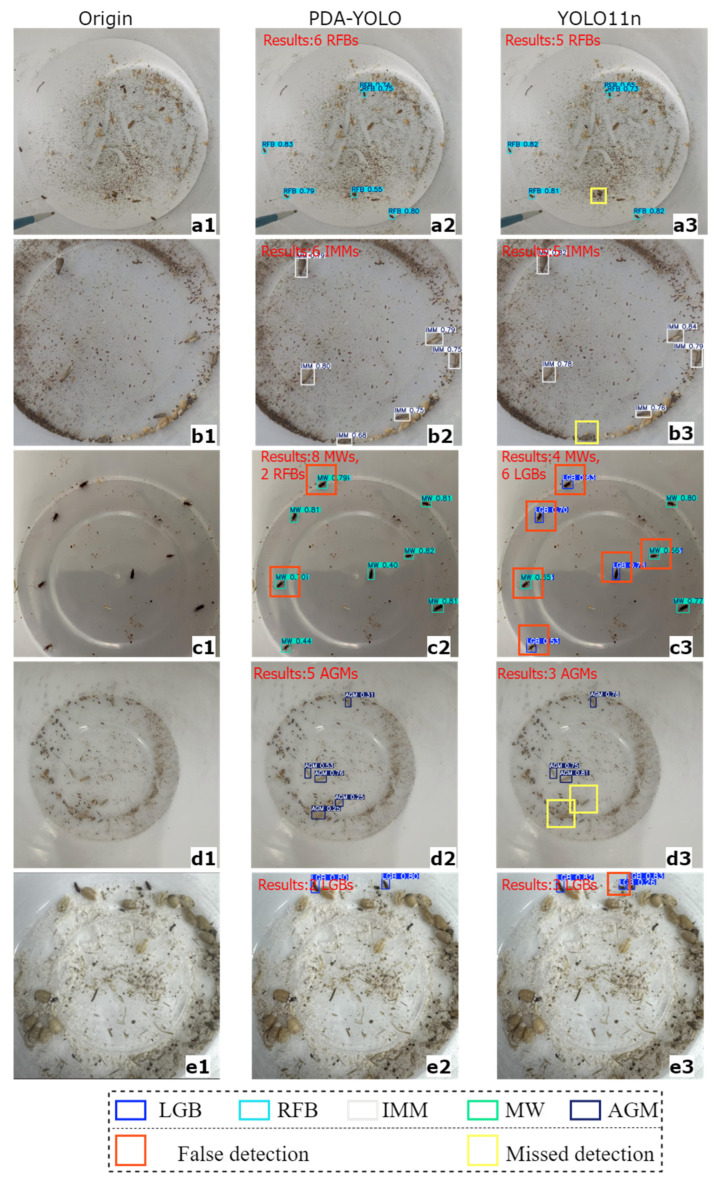
Comparison of detection results. (**a**–**e**) Five representative test samples showing: (**a1**–**e1**) original images; (**a2**–**e2**) PDA-YOLO detection results; (**a3**–**e3**) YOLOv11n detection results.

Based on the comparison of detection results on the test set (Figure 7), this section presents a comprehensive analysis the performance of PDA-YOLO and YOLO11n in stored-grain insect pest detection tasks. First, in detection results of groups a, b, and d (Figure 7), YOLO11n exhibits detection limitations, consistently failing to identify all pest specimens present in the images. In contrast, PDA-YOLO notably reduces false negative rates, successfully detecting pests even in challenging scenarios where pest specimens are easily confused with environmental backgrounds or only partially visible. Second, YOLO11n frequently generates false positives by misidentifying non-target areas (such as grain textures and debris) as pest specimens in complex backgrounds. This limitation is particularly pronounced in the detection of LGB (groups e in Figure 7), where background elements with similar visual characteristics to the target pests are incorrectly classified as positive detections. Conversely, PDA-YOLO shows superior discrimination capability in complex backgrounds, effectively suppressing false positives while maintaining high detection sensitivity. Notably, YOLO11n demonstrates taxonomic misclassification issues in multi-category detection tasks. For example, when detecting MW specimens (group c in Figure 7), YOLO11n frequently misidentifies them as LGB or RFB, resulting in compromised taxonomic precision. In comparison, PDA-YOLO exhibits considerably improved taxonomic accuracy, with substantially fewer misclassification instances across all pest categories, particularly for morphologically similar species, such as the three species of beetles mentioned above.

### 4.4. Grad-CAM

Identifying regions of attentional prominence during model inference elucidates the feature detection tendencies that remain concealed in the non-transparent algorithmic processing pipeline. To further validate the effectiveness of PDA-YOLO in stored-grain insect pest detection, we employed Gradient-weighted Class Activation Mapping (Grad-CAM) to generate and analyze attention heat maps for both YOLO11n and PDA-YOLO. Grad-CAM heat maps utilize visualization techniques to demonstrate the degree of attention that different models allocate to key regions during object detection tasks [36].

**Figure 8:**
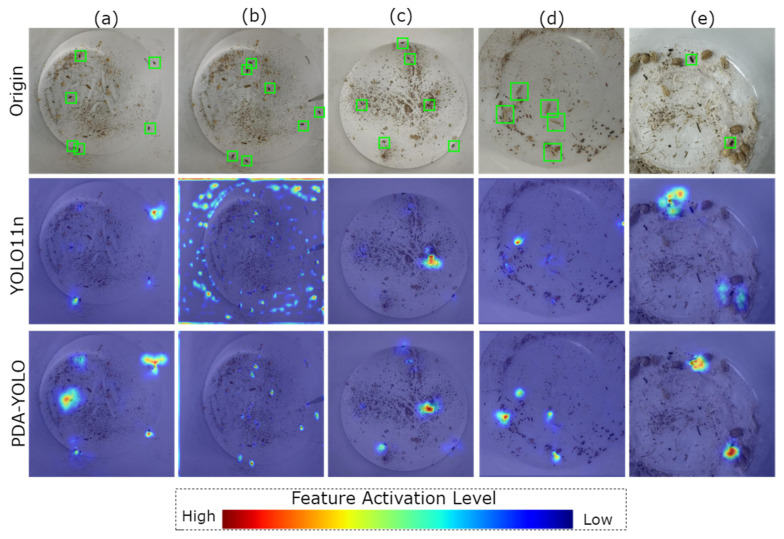
Feature heatmap of stored-grain insect pests for YOLO11n and PDA-YOLO. Subfigures (**a**–**e**) represent five different test cases. For each case, the top row shows the original image with pest locations marked by green bounding boxes, the middle row displays YOLO11n activation heatmaps, and the bottom row shows PDA-YOLO activation heatmaps.

In Figure 8, warm-colored regions (red and yellow) indicate areas with major contributions to detection results, while cool-colored regions (green and blue) represent areas of lesser contribution. In all groups, PDA-YOLO consistently exhibits higher activation intensity in pest target regions compared to YOLO11n, indicating more focused attention on critical features (Figure 8). This improved focus explains PDA-YOLO’s superior detection accuracy, especially for challenging cases where pests are partially occluded or share similar coloration with the background. Additionally, as shown in Figure 8, in groups b and e, YOLO11n displays relatively high activation in non-target background regions, suggesting excessive attention to irrelevant environmental features. In contrast, PDA-YOLO features more selective activation patterns, concentrating primarily on pest specimens while effectively suppressing background interference, which explains its lower false positive rate in complex grain storage environments.

## 5. Discussion

Deep learning technology shows promising potential in intelligent grain storage management. Our proposed PDA-YOLO algorithm, through its three improved modules, achieved exceptional evaluation metrics. The *F*1 score reached 93.5%, mAP@0.5 achieved 96.6%, and mAP@0.5:0.95 attained 60.4%, with a computational cost of only 6.9 G and *mD_t_* of 9.9 ms. These results surpass previous approaches such as AC-YOLO, which reported 91.9% mAP@0.5 and 37.7% mAP@0.5:0.95 for multi-category (specifically 12 species) pest detection [24]. Moreover, AC-YOLO’s higher computational requirements are not conducive to deployment on edge devices. YOLO-TP focused on single-species detection of *Lasioderma serricorne* with comparable accuracy but limited taxonomic scope compared to our multi-species approach [25]. When comparing detection performance across pest categories, PDA-YOLO demonstrates balanced excellence with high detection accuracy exceeding 94% mAP@0.5 across three beetles and two moth species. This balanced performance across various pest morphologies is essential for practical deployment in grain storage facilities where multiple pest species often coexist. In contrast, Shi et al.’s detection method shows accuracy discrepancies among different stored-grain insect pest species [37], with their highest detection accuracy reaching 93.71%, while their lowest was only 83.24%, representing a substantial 10.47% gap.

Modern detection approaches for stored-grain insect pests include acoustic detection systems, NIRS, and electronic nose technology. Mankin et al. [12] developed a low-cost acoustic insect detector system that could detect infestations as low as 1.9 insects/kg for *Sitophilus oryzae* in grain and 3.8 insects/kg for *Tribolium castaneum* in flour. While effective, acoustic methods are susceptible to environmental noise interference and require relatively quiet conditions for optimal performance. Near-infrared spectroscopy, as reviewed by Johnson et al. [13], demonstrates potential for non-invasive detection, particularly at the single-kernel level with detection accuracy of 90–100%. However, NIRS suffers from limitations in bulk sample analysis and requires expensive equipment with specialized calibration procedures. Zhou et al. [16] investigated electronic nose technology for detecting rice infestations, achieving discrimination between clean and infested rice after four weeks of storage at 30 °C, with high accuracy. Nevertheless, electronic nose performance is strongly influenced by storage conditions and may not provide reliable results during early infestation stages. Compared to these approaches, our PDA-YOLO offers several advantages: (1) higher detection accuracy across multiple pest species, without requiring controlled environments, (2) real-time performance for continuous monitoring, and (3) scalability for deployment on edge devices. Additionally, computer vision-based approaches, such as the proposed PDA-YOLO, can monitor larger areas simultaneously and integrate with existing camera infrastructure, making them more cost effective for widespread implementation.

Despite these promising results, limitations exist in the current study. The algorithm is currently limited to five common stored-grain insect pest species within controlled collection bottle environments, restricting broader applicability. Trap-based detection inherently suffers from variable capture efficiency across species and limited spatial coverage. Additionally, while our detection accuracy is impressive, there remains room for improvement in mAP@0.5:0.95 metrics. The current value is 60.4%, indicating challenges in achieving precise bounding box localization across stricter IoU thresholds, which is similar to the findings reported by Zhao et al. and Tian et al. [24,38]. Future research should focus on expanding taxonomic diversity, developing transfer learning techniques for different grain types and detection contexts, implementing domain adaptation for bulk grain inspection, and integrating multi-modal sensing approaches to overcome these limitations and enhance practical deployment capabilities.

## 6. Conclusions

In this study, we proposed PDA-YOLO, an efficient and lightweight algorithm for stored-grain insect pest detection, addressing key challenges including small target recognition, complex background interference, and balancing accuracy with efficiency. The PDA-YOLO algorithm integrates PF_C3k2 and AIFI modules, greatly enhancing feature extraction capabilities and global context awareness. Additionally, the introduction of the DMAE module effectively improves the precision of boundary delineation for tiny targets. Experimental results demonstrated that PDA-YOLO exhibits advantages in achieving an optimal balance between detection accuracy and computational efficiency compared to both YOLO-series and other mainstream object detection algorithms. On our custom stored-grain insect pest dataset, PDA-YOLO achieved mAP@0.5 and mAP@0.5:0.95 scores of 96.6% and 60.4%, respectively, with a computational cost of only 6.9 G and *mD_t_* of 9.9 ms. The algorithm provides valuable technical support for the development of intelligent grain storage management systems and contributes to advancing food security protection measures.

## Data Availability

The dataset used in this study is not publicly available due to laboratory policy restrictions. The dataset containing stored-grain insect pest images and annotations remains proprietary to the Key Laboratory of Grain Information Processing and Control at Henan University of Technology. Reasonable requests for data access may be considered on a case-by-case basis and should be directed to the corresponding author, subject to institutional approval and appropriate confidentiality agreements.
